# Demenzprädiktion als ethische Herausforderung: Stakeholder fordern Beratungsstandards für Deutschland

**DOI:** 10.1007/s00115-020-00985-y

**Published:** 2020-08-28

**Authors:** Silke Schicktanz, Julia Perry, Benjamin Herten, Scott Stock Gissendanner

**Affiliations:** 1 Institut für Ethik und Geschichte der Medizin, Universitätsmedizin Göttingen, Humboldtallee 36, 37073 Göttingen, Deutschland; 2 IEGUS - Institut für europäische Gesundheits- und Sozialwirtschaft, Gesundheitscampus-Süd 29, Bochum, 44801 Deutschland; 3 Sozialwissenschaftliche Fakultät, Georg-August-Universität Göttingen, Platz der Göttinger Sieben 3, Göttingen, 37073 Deutschland

## Hintergrund

Neue biomarkerbasierte Bluttests mit hoher Sensitivität und Spezifizität für die Prädiktion einer Alzheimer-Demenz bei symptomfreien Personen sind aktuell in der Entwicklung [1–3]. Biomarker zur Alzheimer-Früherkennung, beispielsweise mittels Liquorbiomarker und Amyloid-Positronenemissionstomographie (Amyloid-PET), kommen heute in der Klinik bereits zum Einsatz, auch in Form eines anlasslosen „Brain-Checks“ für Zielgruppen ohne medizinische Indikation [4]. Selbst in Fällen, bei denen diese Verfahren medizinisch indiziert sind, sind sie verhältnismäßig teuer und invasiv. Wenn in naher Zukunft kostengünstigere und weniger invasive Prädiktionstests zur Verfügung stehen, was erwartet wird [5], stellen sich ungeklärte Fragen, mit denen sich Betroffene, die Gesellschaft und vor allem beratende Personen intensiv auseinandersetzen müssen.

Die klinische Anwendung solcher Testverfahren für Menschen ohne objektivierbare Symptome birgt rechtliche, ethische und soziale Probleme [6], die bis heute noch nicht ausreichend diskutiert oder annährend gelöst worden sind. Die Stellungnahme der Bundesärztekammer vom März 2018 zur prädiktiven Demenzdiagnostik [7] stellt einen wichtigen Schritt im gesellschaftlichen und gesundheitspolitischen Diskurs dar. Sie bezieht sich jedoch nicht auf die zukünftig zu erwartende Einführung blutbasierter Testverfahren zur Prädiktion bei asymptomatischen Personen, welche mit einem entsprechend erhöhten allgemeinen Beratungsbedarf sowie erheblichen gesellschaftlichen Auswirkungen einhergeht. Es besteht daher ein dringender gesellschaftlicher Konsentierungsbedarf zu diesen ungelösten Fragen.

## Der Diskurs zum Umgang mit künftigen medizinethischen Herausforderungen

Vorausschauende Governance setzt auf einen Diskurs, der die möglichen Auswirkungen dieser zukünftigen Technologie frühzeitig antizipiert und versucht, ethisch-rechtliche Lösungsansätze in Form von Empfehlungen und Richtlinien zu entwerfen. Von der Nutzung solcher prädiktiver Testverfahren sind unterschiedliche Personengruppen betroffen. Diese sollten ihre Interessen frühzeitig definieren können und in die gesundheitspolitischen Entscheidungsprozesse einbringen. Um dies zu ermöglichen, wurde von Oktober 2017 bis September 2019 das vom Bundesministerium für Bildung und Forschung (BMBF) geförderte Diskursverfahren „Konfliktfall Demenzvorhersage“ (Förderkennzeichen: 01GP1770 A, B) durchgeführt. Das Projekt beförderte die Diskussion zu potenziellen Auswirkungen der Demenzprädiktion im deutschen Kontext unter den vielfältigen Berufsverbänden, Selbsthilfe- und Fachorganisationen, welche Entscheidungen über prädiktive Demenzdiagnostik treffen oder deren Mitglieder von gesellschaftlichen und gesundheitspolitischen Entscheidungen in Bezug auf Demenz, Gesundheitsversorgung oder Altersvorsorge betroffen sind. In der Definition der Projektmethode sind dies die gesellschaftlichen „Stakeholder“. Somit wurden als Diskursteilnehmer gezielt organisierte Interessensvertretungen der Zivilgesellschaft anvisiert. Das Verfahren gab ihnen Anlass, ihre Interessen hervorzuheben und sich mit anderen Stakeholdern auszutauschen, um eine gemeinsame Position zu entwickeln.

Für die diskursive Beteiligung wurde im Projekt ein iteratives, mehrstufiges Verfahren entwickelt. Vier aufeinander aufbauende Phasen konnten einzeln oder gänzlich von jeder Organisation in Anspruch genommen werden: schriftlicher Input, Konferenzteilnahme, Kommentierung und Kenntnisnahme. Zum Auftakt des Projekts wurden 80 Organisationen aufgefordert, mit einem einseitigen „Problemaufriss“ am Diskursverfahren teilzunehmen. 29 Organisationen reichten schriftliche Positionspapiere ein. 24 dieser Organisationen benannten Delegierte für die Teilnahme an einer zweitägigen, intensiven Konferenz zur Konsentierung einer gemeinsamen Stellungnahme (22. bis 23. Juni 2018 in Göttingen). Damit ist erstmals in Deutschland eine gemeinsame Position geschaffen worden, aus der sich der fachliche und öffentliche Diskurs weiterentwickeln kann. Die gemeinsame Stellungnahme wurde in einer redaktionell überarbeiteten Endversion Ende Dezember 2018 veröffentlicht und allen Stakeholdern zur Kommentierung vorgelegt [8]. Die Positionspapiere, die gemeinsame Stellungnahme und die Kommentare sind auf der Projektwebsite veröffentlicht (www.praediadem.de). Alle Organisationen, die sich in das Verfahren eingebracht haben, sind in Tab. 1 aufgelistet.

**Table 1:** 

Akademie für Ethik in der Medizin (AEM; AG Altern und Ethik/AG Ethik und Pflege II)
Arbeiterwohlfahrt (AWO) Bundesverband
Bundesapothekerkammer (BAK)
Bundesarbeitsgemeinschaft der Seniorenorganisationen (BAGSO)
Bundesinitiative Ambulante Psychiatrische Pflege (BAPP)
Demenz Support Stuttgart
Deutsche Alzheimer Gesellschaft (DAlzG)
Deutsche Bischofskonferenz (DBK)
Deutscher Berufsverband für Soziale Arbeit (DBSH)
Deutsche Gesellschaft für Allgemeinmedizin und Familienmedizin (DEGAM)
Deutsche Gesellschaft für Gerontologie und Geriatrie (DGGG)
Deutsche Gesellschaft für Gerontopsychiatrie und -psychotherapie (DGGPP)
Deutsche Gesellschaft für Liquordiagnostik und Klinische Neurochemie (DGLN)
Deutsche Gesellschaft für Neurologie (DGN)
Deutsche Gesellschaft für Pflegewissenschaft (DGP)
Deutsche Gesellschaft für Psychiatrie und Psychotherapie, Psychosomatik und Nervenheilkunde (DGPPN)
Deutscher Gewerkschaftsbund (DGB)
Deutsches Netzwerk Evidenzbasierte Medizin (DNEbM)
Deutscher Pflegerat (DPR)
Deutsche Vereinigung für Soziale Arbeit im Gesundheitswesen (DVSG)
Deutsches Zentrum für Neurodegenerative Erkrankungen (DZNE) – Klinische Forschung
Diakonie Deutschland & Zentrum für Gesundheitsethik (ZfG)
Dialog- und Transferzentrum Demenz (DZD) Witten
Gesellschaft für Neuropsychologie (GNP)
Institut für Qualität und Wirtschaftlichkeit im Gesundheitswesen (IQWiG)
Institut für Technikfolgenabschätzung und Systemanalyse (ITAS)
Kassenärztliche Bundesvereinigung (KBV)
Landesinitiative Demenz-Service Nordrhein-Westfalen (LID)
Spitzenverband ZNS (SpiZ)
Verband der Ersatzkassen (vdek)
Verband der Privaten Krankenversicherung (PKV)
Verband Forschender Arzneimittelhersteller (vfa)
Zentralrat der Muslime in Deutschland (ZMD)

Das Diskursverfahren hat methodisch wie inhaltlich zeigen können, dass es möglich ist, viele und durchaus heterogen ausgerichtete Stakeholder unter ökonomischen und zeitlichen Limitationen einzubinden und gemeinsame Positionen zu komplexen ethischen Fragestellungen entwickeln zu lassen. Das Projekt hat mit dieser Methode eine neue Form des gesellschaftlichen und gesundheitspolitischen Diskurses gestaltet.

## Forderungen der Stakeholder im Diskursverfahren

Die gemeinsame Stellungnahme zeichnet sich dadurch aus, dass sie zurzeit im deutschsprachigen Raum die einzige konsensuelle, multiprofessionelle und spartenübergreifende Position zum zukünftigen Umgang mit der neuen Technologie der Demenzprädiktion darstellt. Die gemeinsame Stellungnahme benennt konkret fünf zentrale Desiderate. Diese müssen aus Sicht der deutschen Stakeholder adressiert werden, um potenziellen individuellen Schaden und gesellschaftliche Nachteile im Zuge einer Verbreitung prädiktiver Testverfahren zu minimieren. Werden diese Desiderate angemessen adressiert, kann dies aus Sicht der Projektleitung dazu verhelfen, auch befürwortenden Argumenten zur Prädiktion, die einen potenziellen Nutzen und das Recht auf Wissen beinhalten, in ausbalanciertem Maße Rechnung zu tragen. Werden diese Desiderate allerdings nicht adressiert, kann dies zu extremen Formen der Ablehnung führen, wie z. B. Forderungen nach einem Moratorium.

1. Es sollen neue Impulse für die teilhabeorientierte gesellschaftliche Verständigung über das Leben mit Demenz begleitend zur Verbreitung prädiktiver Testverfahren angeregt werden. Aus Sicht der Projektleitung ist dies ein Aspekt für die nationale Demenzstrategie (www.nationale-demenzstrategie.de).
2. Die individuellen und sozialen Auswirkungen der Prädiktion müssen systematisch erforscht werden. Als Forschungsförderer sind hier nicht nur das Bundesministerium für Bildung und Forschung sowie das Bundesgesundheitsministerium gefragt, sondern auch private Forschungsstiftungen.
3. Leitlinien für die technische Umsetzung der Verfahren für Labore und Kliniken sind notwendig. Einschlägige Fachgesellschaften sollten dies initiieren. Ringversuchsinitiativen und Zertifizierungen, die im Bereich Liquordiagnostikverfahren zur Routine gehören, sollten für prädiktive Testverfahren mittels Biomarker erweitert werden.
4. Es bedarf einer Vereinbarung für die Bestimmung, wer als Anbieter von Biomarkertests befugt werden sollte und wer für die Beratung von Testprobanden im klinischen Bereich verantwortlich ist.
5. Es wird dringend eine Leitlinienentwicklung bzw. Rahmenvereinbarung für eine qualitativ hochwertige Beratung vor und nach Durchführung eines Testverfahrens gefordert. Das Diskursverfahren „Konfliktfall Demenzvorhersage“ macht deutlich, dass für die produktive Zusammenarbeit nicht einzelne Fachgesellschaften oder Fachkommissionen zuständig sein können. Die Zukunft liegt darin, dass ethische Empfehlungen und Leitlinien, die Beratungsstandards betreffen, durch die Fachgesellschaften mehrerer Berufsgruppen entwickelt werden und die Bereiche Medizin, Pflege, psychosoziale Beratung, Ethik, Recht und insbesondere Betroffenenverbände einbezogen werden. Die Methode des breiten Diskursverfahrens hat den Vorteil, dass Wert- und Interessenskonflikte frühzeitig deutlich gemacht und zugleich Konsensbereiche herausgearbeitet werden, die dann für die Entwicklung klinischer Leitlinien als Ausgangsmotivation dienen können.

Die Forderungen der Stakeholder stimmen zum größten Teil mit den Forderungen und Empfehlungen aus dem aktuellen Stand des internationalen wissenschaftlichen Ethikdiskurses überein [9–11]. Trotz der Entwicklung und Implementierung nationaler Demenzstrategien in anderen Ländern fehlen auch in diesen Ländern konkrete Leitlinien zum Umgang mit und zur Beratung bei der Demenzprädiktion. Die uns bekannten, bisher existenten Leitlinien in den USA, Deutschland und Kanada fokussieren auf die Verbesserung der Diagnosestellung sowie auf die Erstellung eines Forschungsplans für die Früherkennung [12].

Im US-Kontext bieten beispielsweise die „Appropriate Use Criteria (AUC)“ zu Amyloid-PET-Verfahren einen 5‑Phasen-Plan zur Implementierung an [13], welcher größtenteils den Forderungen der Stakeholder in Deutschland für den Einsatz biomarkerbasierter Bluttests entspricht. Unser Diskursverfahren will als Anstoß dienen, die aktuellen Lücken bezüglich Beratung im Kontext nichtgenetischer Prädiktion einer Alzheimer-Demenz zu schließen.

## Fazit für die Praxis

- Bluttests für die Prädiktion einer Alzheimer-Demenz werden wahrscheinlich zukünftig in der Forschung und im klinischen Bereich angewendet.
- Das Diskursverfahren „Konfliktfall Demenzvorhersage“ ergab eine gemeinsame Stellungnahme deutscher Verbände und Fachgesellschaften zu den praktischen und ethischen Implikationen der prädiktiven Demenzdiagnostik (www.praediadem.de) mit fünf Forderungen: neue Impulse für eine teilhabeorientierte Verständigung über das Leben mit Demenz, Erforschung von Auswirkungen der Prädiktion, Leitlinien für Labore, eine Vereinbarung in der Frage, wer als Testanbieter befugt werden sollte, und eine Leitlinie für die Beratung.
